# Characterization of host and *escherichia coli* strains causing recurrent urinary tract infections based on molecular typing

**DOI:** 10.1186/s12866-023-02820-1

**Published:** 2023-03-30

**Authors:** Cheng-Yen Kao, Yen-Zheng Zhang, Deng-Chi Yang, Pek Kee Chen, Ching-Hao Teng, Wei-Hung Lin, Ming-Cheng Wang

**Affiliations:** 1 Institute of Microbiology and Immunology, College of Life Sciences, National Yang Ming Chiao Tung University, Taipei, Taiwan; 2 Department of Geriatrics and Gerontology, National Cheng Kung University Hospital, College of Medicine, National Cheng Kung University, Tainan, Taiwan; 3 Institute of Molecular Medicine, College of Medicine, National Cheng Kung University, Tainan, Taiwan; 4 Institute of Basic Medical Sciences, College of Medicine, National Cheng Kung University, Tainan, Taiwan; 5 Center of Infectious Disease and Signaling Research, National Cheng Kung University, Tainan, Taiwan; 6 Department of Internal Medicine, National Cheng Kung University Hospital, College of Medicine, National Cheng Kung University, Tainan, Taiwan; 7 Division of Nephrology, Department of Internal Medicine, National Cheng Kung University Hospital, College of Medicine, National Cheng Kung University, No.1, University Road, Tainan City, 701 Taiwan

**Keywords:** Recurrent urinary tract infection, *Escherichia coli*, Pulsed-field gel electrophoresis, Virulence, Antimicrobial resistance

## Abstract

**Background:**

*Escherichia coli* is the leading pathogen responsible for urinary tract infection (UTI) and recurrent UTI (RUTI). Few studies have dealt with the characterization of host and bacteria in RUTI caused by *E. coli* with genetically identical or different strains. This study aimed to investigate the host and bacterial characteristics of *E. coli* RUTI based on molecular typing.

**Results:**

Patients aged 20 years or above who presented with symptoms of UTI in emergency department or outpatient clinics between August 2009 and December 2010 were enrolled. RUTI was defined as patients had 2 or more infections in 6 months or 3 or more in 12 months during the study period. Host factors (including age, gender, anatomical/functional defect, and immune dysfunction) and bacterial factors (including phylogenicity, virulence genes, and antimicrobial resistance) were included for analysis. There were 41 patients (41%) with 91 episodes of *E. coli* RUTI with highly related PFGE (HRPFGE) pattern (pattern similarity > 85%) and 58 (59%) patients with 137 episodes of *E. coli* RUTI with different molecular typing (DMT) pattern, respectively. There was a higher prevalence of phylogenetic group B2 and *neuA* and *usp* genes in HRPFGE group if the first episode of RUTI caused by HRPFGE *E. coli* strains and all episodes of RUTI caused by DMT *E. coli* strains were included for comparison. The uropathogenic *E. coli* (UPEC) strains in RUTI were more virulent in female gender, age < 20 years, neither anatomical/ functional defect nor immune dysfunction, and phylogenetic group B2. There were correlations among prior antibiotic therapy within 3 months and subsequent antimicrobial resistance in HRPFGE *E. coli* RUTI. The use of fluoroquinolones was more likely associated with subsequent antimicrobial resistance in most types of antibiotics.

**Conclusions:**

This study demonstrated that the uropathogens in RUTI were more virulent in genetically highly-related *E. coli* strains. Higher bacterial virulence in young age group (< 20 years) and patients with neither anatomical/functional defect nor immune dysfunction suggests that virulent UPEC strains are needed for the development of RUTI in healthy populations. Prior antibiotic therapy, especially the fluoroquinolones, within 3 months could induce subsequent antimicrobial resistance in genetically highly-related *E. coli* RUTI.

**Supplementary Information:**

The online version contains supplementary material available at 10.1186/s12866-023-02820-1.

## Background

Urinary tract infection (UTI) is a common infectious disease in the urinary tract. Nearly half of all women experience a UTI in their lifetime, and up to 27%-50% of these patients will have a recurrent infection in the following 6 months [1–3]. Recurrent UTI (RUTI) occurs due to bacterial persistence or bacterial reinfection, and *Escherichia coli* is one of the dominant pathogens responsible for RUTI [4]. Bacterial persistence is defined by the same bacteria strain not being eradicated within the host 2 weeks after antibiotic treatment. Reinfection is a recurrence with a different microorganism, the same microorganism in more than 2 weeks, or a sterile intervening culture [5].

The increasing prevalence and growing problem of antibiotic resistance among uropathogens present a critical challenge to the clinical management of RUTI [6].

There are three possible mechanisms responsible for correctly treated infections with subsequent gain of resistance: evolution of resistance through mutations, through dedicated resistance genes, and through reinfection with a different strain resistant to antibiotics [7]. Prior antimicrobial drug exposure is a risk factor for resistant UTI, especially after receiving multiple courses of antibiotics for recurrent infections [8, 9].

There were several studies investigating the bacterial characteristics in RUTI caused by uropathogenic *E. coli* (UPEC) with genetically identical or different strains. Regarding the RUTI in adults with community-acquired pyelonephritis caused by *E. coli*, Kärkkäinen et al. reported that genotype comparisons by random amplified polymorphic DNA (RAPD)-PCR analysis showed that 75% of the original and recurrent strains were genetically non-identical. Virulence factors were evenly distributed among *E. coli* isolates of index episodes, independent of the recurrences. Lindblom et al. reported that half of the patients with *E. coli* RUTI were infected with ST131 isolates, and Clade C2 were the dominant subsets among ST131 isolates and more common in patients with RUTI than sporadic UTI [10, 11]. The aims of this study were to investigate the host characteristics, bacterial virulence, and antimicrobial resistance in genetically highly-related and genetically discordant *E. coli* strains of RUTI based on molecular typing.

## Results

**Fig. 1:**
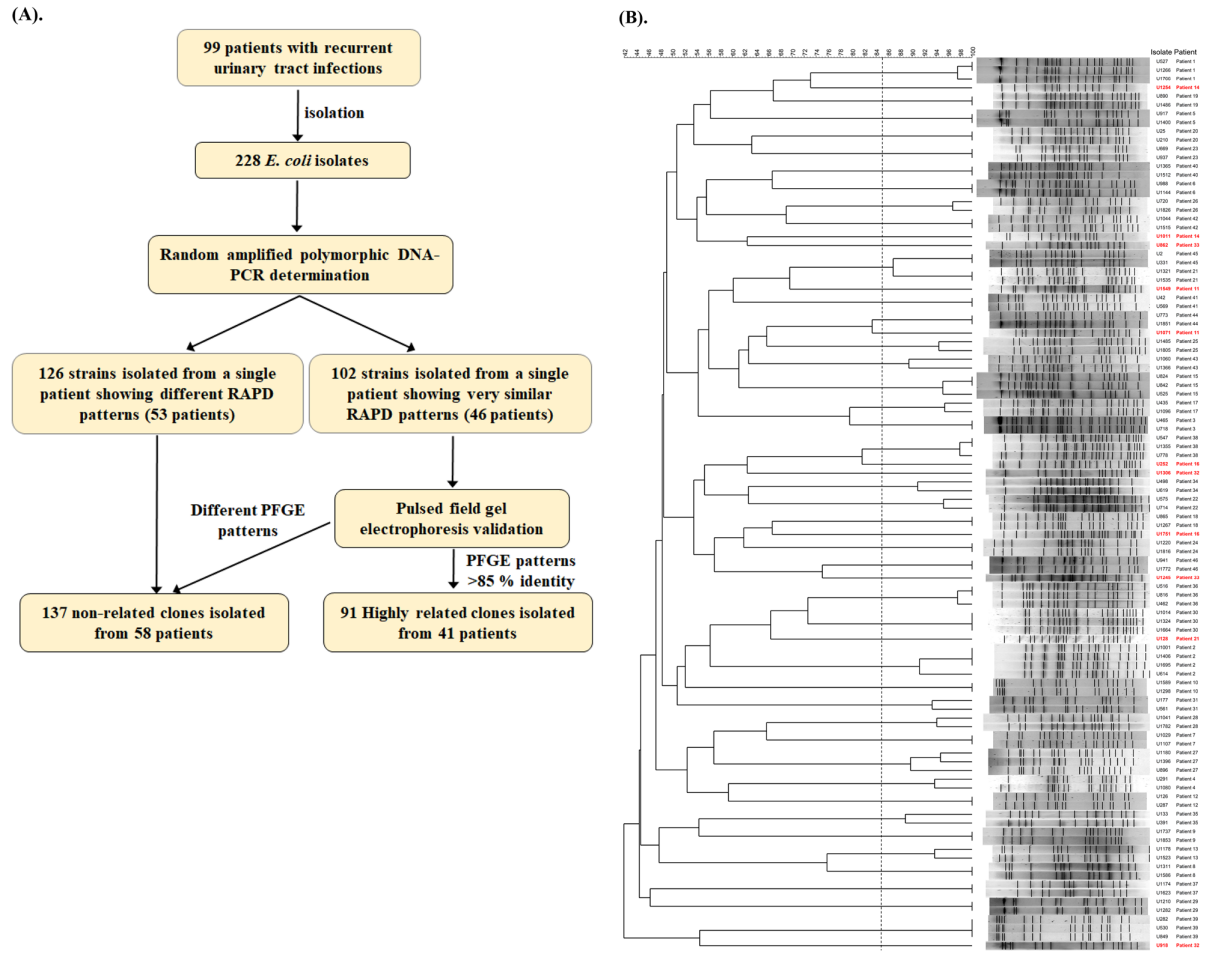
**PFGE analysis to determine the clonality of*E. coli*strains isolates from 99 patients with recurrent UTI. (A).** Experimental flow chart procedures of *E. coli* collection and clonality determination. **(B).** PFGE patterns of 102 strains isolated from 46 RUTI patients. Eleven strains shown in red were considered as negative controls with different PFGE patterns. The black dotted line is the 85% similarity line.

A total of 99 patients with 228 episodes of RUTI (including the first episode) caused by *E. coli* were included for analysis (Fig. 1A). Four primers, namely 1247, 1254, 1283, and 1290, were used in the time-saving and cost-saving random amplification of polymorphic DNA (RAPD)-PCR assay to determine the clonality of *E. coli* isolated from a single patient. The results showed that 46 of 99 RUTI patients (a total of 102 isolates) were suspected to be infected by the closely related clones (Fig. 1A). Pulsed-field gel electrophoresis (PFGE) was performed on 102 strains isolated from 46 patients to validate RAPD-PCR results (Fig. 1B). Strains isolated from a single patient showing PFGE patterns > 85% identity with a tolerance of 0.9% and an optimization parameter of 0.9% by GelCompar II software were defined as highly related strains (Fig. 1B). There were 41 patients (41%) with 91 episodes of *E. coli* RUTI with highly related PFGE (HRPFGE) pattern and 58 (59%) patients with 137 episodes of *E. coli* RUTI with different molecular typing (DMT) pattern, respectively (Fig. 1A & 1B). Interestingly, three UPEC strains (U128, U1321, U1535) with two PFGE patterns were isolated from patient 21. Female gender was predominant (74%). The bacterial characteristics in relation to molecular typing grouping in patients with RUTI are shown in Table 1. There was no significant difference in phylogenicity and bacterial virulence between HRPFGE and DMT *E. coli* strains in first episode of RUTI. If first episode of RUTI caused by HRPFGE *E. coli* strains and all episodes of RUTI caused by DMT *E. coli* strains were included for comparison, there was a higher prevalence of phylogenetic group B2 and *neuA* and *usp* genes in HRPFGE group. The host characteristics in relation to molecular typing grouping in 99 patients with *E. coli* RUTI are shown in Table 2. There was no significant difference in age, anatomical/functional defect, or immune dysfunction between PFGE identical and molecular typing different groups; there was a higher prevalence of male gender in the HRPFGE group.

**Table 1:** Bacterial characteristics in relation to molecular typing grouping in patients with recurrent urinary tract infection (total 178 isolates)

Characteristic	Highly related PFGE pattern, first episode (n = 41) n (%)	Different molecular typing pattern, first episode (n = 58) n (%)	Different molecular typing pattern, all episodes (n = 137) n (%)	*P-*value ^a^	*P*-value ^b^
Phylogenetic group				0.0163	0.0003
A	0 (0)	4 (7)	12 (9)		
B1	1 (2)	9 (16)	28 (20)		
B2	28 (68)	27 (47)	59 (43)		
D	10 (24)	18 (31)	38 (28)		
Untypable	2 (5)	0	0		
Virulence factor					
*neuA*	12 (29)	9 (16)	19 (14)	0.1346	0.0332
*papG I*	0	0	0	--	--
*papG II*	8 (20)	12 (21)	25 (18)	1.0000	0.8223
*papG III*	5 (12)	9 (16)	19 (14)	0.7732	1.0000
*sfa*	2 (5)	4 (7)	7 (5)	1.0000	1.0000
*foc*	2 (5)	5 (9)	8 (6)	0.6962	1.0000
*cnf1*	7 (17)	8 (14)	15 (11)	0.7776	0.2903
*aer*	27 (66)	40 (69)	86 (63)	0.8284	0.8536
*usp*	28 (68)	28 (48)	60 (44)	0.0642	0.0074
*iha*	16 (39)	13 (22)	36 (26)	0.1157	0.1220
*ompT*	33 (80)	42 (72)	88 (64)	0.4761	0.0575
*afa*	14 (34)	30 (52)	56 (41)	0.1022	0.4716
*iroN*	11 (27)	24 (41)	51 (37)	0.2001	0.2644
*fimH*	39 (95)	55 (95)	126 (92)	1.0000	0.7352
*hlyA*	11 (27)	8 (14)	19 (14)	0.1248	0.0600
*sat*	15 (37)	12 (21)	35 (26)	0.1089	0.1720
PFGE: pulsed-field gel electrophoresis ^a^ Highly related PFGE pattern, first episode versus different molecular typing pattern, first episode ^b^ Highly related PFGE pattern, first episode versus different molecular typing pattern, all episodes					

**Table 2:** Host characteristics in relation to molecular typing grouping in 99 patients with recurrent urinary tract infection (first episode)

Characteristic	Highly related PFGE pattern (n = 41) n (%)	Different molecular typing pattern (n = 58) n (%)	*P*-value
Age (year)	64 ± 24	59 ± 26	0.3061
Gender (male)	18 (44)	8 (14)	0.0011
Anatomical/functional defect	18 (44)	21 (36)	0.5320
Immune dysfunction	16 (39)	29 (50)	0.3111
Both anatomical/functional defects and immune dysfunction	6 (15)	5 (9)	0.5178
Neither anatomical/functional defect nor immune dysfunction	13 (32)	13 (22)	0.3568

PFGE: pulsed-field gel electrophoresis

The bacterial characteristics in relation to gender in 99 RUTI patients showed no difference in phylogenicity or virulence genes (Table S1). The bacterial characteristics in relation to age in 99 RUTI patients showed a higher prevalence of *foc* and *cnf1* genes in the age < 20 years group (Table S2). The bacterial characteristics in relation to anatomical/ functional defect and immune dysfunction in 99 RUTI patients showed a higher prevalence of *papG III, sfa*, and *hlyA* genes in neither anatomical/ functional defect nor immune dysfunction group (Table 3). The bacterial characteristics in relation to phylogenetic group B2 in 99 RUTI patients showed a higher prevalence of *neuA*, *sfa, cnf1*, *usp, iha, ompT, afa, hlyA*, and *sat* genes (Table 4).

**Table 3:** Bacterial characteristics in relation to anatomical/ functional defect and immune dysfunction in 99 patients with recurrent urinary tract infection (first episode)

Characteristic	Either anatomical/ functional defect or immune dysfunction (n = 73) n (%)	Neither anatomical/ functional defect nor immune dysfunction (n = 26) n (%)	*P*-value
Phylogenetic group			0.6586
A	3 (4)	1 (4)	
B1	8 (11)	2 (8)	
B2	38 (52)	17 (65)	
D	23 (32)	5 (19)	
Untypable	1 (1)	1 (4)	
Virulence factor			
*neuA*	14 (19)	7 (27)	0.4136
*papG I*	0	0	--
*papG II*	16 (22)	4 (15)	0.5784
*papG III*	6 (8)	8 (31)	0.0084
*sfa*	2 (3)	4 (15)	0.0396
*foc*	3 (4)	4 (15)	0.0752
*cnf1*	9 (12)	6 (23)	0.2110
*aer*	52 (71)	15 (58)	0.2285
*usp*	39 (53)	17 (65)	0.3596
*iha*	21 (29)	8 (31)	1.0000
*ompT*	52 (71)	23 (88)	0.1099
*afa*	36 (49)	8 (31)	0.1142
*iroN*	22 (30)	13 (50)	0.0944
*fimH*	70 (96)	24 (92)	0.6043
*hlyA*	10 (14)	9 (35)	0.0389
*sat*	20 (27)	7 (27)	1.0000

**Table 4:** Bacterial characteristics in relation to phylogenetic group B2 in 99 patients with recurrent urinary tract infection (first episode)

Characteristic	Phylogenetic group B2 (n = 55) n (%)	Non-phylogenetic group B2 (n = 44) n (%)	*P*-value
Virulence factor			
*neuA*	20 (36)	1 (2)	< 0.0001
*papG I*	0	0	--
*papG II*	15 (27)	5 (11)	0.0766
*papG III*	6 (11)	8 (18)	0.3875
*sfa*	6 (11)	0	0.0322
*foc*	6 (11)	1 (2)	0.1280
*cnf1*	14 (25)	1 (2)	0.0013
*aer*	39 (71)	28 (64)	0.5186
*usp*	52 (95)	4 (9)	< 0.0001
*iha*	25 (45)	4 (9)	< 0.0001
*ompT*	55 (100)	20 (45)	< 0.0001
*afa*	19 (35)	25 (57)	0.0413
*iroN*	22 (40)	13 (30)	0.2990
*fimH*	53 (96)	41 (93)	0.6530
*hlyA*	15 (27)	4 (9)	0.0380
*sat*	24 (44)	3 (7)	< 0.0001

The antimicrobial susceptibility in RUTI related to HRPFGE *E. coli* (41 patients, 91 episodes) is shown in Table S3. There was no significant difference in antimicrobial susceptibility of most antibiotics between the first episode and second episode of RUTI *E. coli* strains. The serial antimicrobial susceptibility in recurrent urinary tract infections related to HRPFGE *E. coli* strains is shown in Table S4. The relationships among prior antibiotic therapy within 3 months and antimicrobial resistance in subsequent 91 episodes of RUTI related to HRPFGE *E. coli*, are shown in Table S5. There were correlations among prior antibiotic therapy within 3 months and subsequent antimicrobial resistance in HRPFGE *E. coli* RUTI, and the use of fluoroquinolones was associated with more antimicrobial resistance of UPEC in the following RUTI. The use of flomoxef, 1st generation cephalosporins, ampicillin or ampicillin/sulbactam, and trimethoprim/sulfamethoxazole was not associated with antimicrobial resistance in all types of antibiotics during the following 3 months. The use of fluoroquinolones was more likely associated with antimicrobial resistance in most types of antibiotics [flomoxef, piperacillin/tazobactam, cephalosporins (1st generation, 2nd generation, and 3rd generation) and fluoroquinolones] during the following 3 months. The use of 2nd generation and 3rd generation cephalosporin was associated with subsequent antimicrobial resistance in flomoxef, and the use of aminoglycosides was associated with subsequent antimicrobial resistance in gentamicin and trimethoprim/sulfamethoxazole during the following 3 months.

## Discussion

RUTI may be caused by repeated ascending infections or chronic/persistent infections in the bladder [1]. *E. coli* is the leading pathogen responsible for RUTI. RUTI may be caused by the same or different *E. coli* strains. There have been several studies presenting the bacterial characteristics (phylogenicity, virulence factors, and biofilm), similarity and difference, and genomic variation in *E. coli* RUTI [10, 12, 13]. However, there have been scarce reports dealing with the host and bacterial characteristics based on the molecular typing in *E. coli* RUTI. Our study demonstrated and compared the patterns of host characteristics and serial bacterial characteristics between genetically highly-related and different *E. coli* strains in RUTI. The UPEC strains in RUTI were more virulent in female gender, age < 20 years, neither anatomical/functional defect nor immune dysfunction, and phylogenetic group B2. In HRPFGE *E. coli* RUTI, there were correlations among prior antibiotic therapy within 3 months and subsequent antimicrobial resistance in HRPFGE *E. coli* RUTI. The use of fluoroquinolones was more likely to have antimicrobial resistance in most types of antibiotic during the following 3 months.

There were several bacterial characteristics contributing to the development of *E. coli* UTI, the phylogenicity, virulence factors, and antimicrobial resistance of UPEC strains varied from region to region [14–19]. The study dealing with uncomplicated community-acquired UTI in women by PFGE showed that 77% after Pivmecillinam treatment had a relapse with the primary infecting *E. coli* strains [20]. Several studies demonstrated the recurrent rate of highly related strains in RUTI varied from 34–82% based on PFGE [6, 21–24]. Nielsen et al. reported that RUTI *E. coli* isolates did not cluster distinct from non-RUTI isolates in a single nucleotide polymorphism (SNP) phylogeny [13]. Our study showed that 41% of UPEC strains in RUTI were generically highly related. Phylogenetic group B2 was the most predominant. There was no significant difference in phylogenicity and virulence profile between HRPFGE and DMT *E. coli* strains in the first episode of RUTI. Whereas increased bacterial virulence was present in HRPFGE *E. coli* strains if all episodes of RUTI are included for comparison.

Repeated ascending infection and chronic/persistent infection in the bladder are the two possible mechanisms of RUTI. It has been suggested that RUTI is a consequence of complex host–pathogen interactions involving bacterial factors and deficiency in host defense [25–27]. Several host factors have been associated with UTI and RUTI, which include anatomic and functional disorders (e.g., female gender, post-menopause, vaginal infection, diabetes, urinary obstruction, urinary retention, immunosuppression, renal failure, renal transplantation, pregnancy, urolithiasis, and indwelling catheters or other drainage devices) [13, 26, 28]. There have been few studies investigating the host characteristics in relation to molecular typing in RUTI. This study showed that there was a higher prevalence of male gender in the HRPFGE group compared to that in the DMT group. Overall, there was no significant difference in phylogenicity and virulence between HRPFGE and DMT groups. There was a significantly higher bacterial virulence (*foc* and *cnf1* genes) in the young age group (< 20 years), and a significantly lower bacterial virulence (*papGIII*, *sfa* and, *iroN* genes) in patients with either anatomical/functional defect or immune dysfunction.

Phylogenetic group B2 prevailed in UPEC strains of UTI and RUTI [6, 16, 17, 29–31]. A study by Ejrnæs et al. showed that *E. coli* isolates causing persistence or relapse were more often of phylogenetic group B2, and were characterized by a higher prevalence of virulence factors. No specific combination of presence/absence of virulence factors could serve as a marker to predict RUTI [12]. Luo et al. reported that the persistence strains had more phylogenetic group B2 and virulence genes than the reinfection strains in *E. coli* RUTI [23]. Our study revealed that phylogenetic group B2 was the most predominant group and harbored more virulence genes in virulence profiles than the other phylogenetic groups in *E. coli* RUTI.

There was an increasing trend in antimicrobial resistance associated with more RUTI episodes [32]. Genomic surveillance of antibiotic-resistant uropathogens shows that drug-resistant clones persisted within and transmitted between the intestinal and urinary tracts of patients affected by RUTI [33]. Among women with recurrent UTI receiving prophylaxis, the susceptibility pattern of *E. coli* strains within one month before a symptomatic *E. coli* UTI could be used to make informed choices for empirical antibiotic treatment [34]. The impact of antimicrobial resistance on the development of RUTI remains controversial.

Luo et al. reported that the antimicrobial susceptibilities of UPEC isolates had little effect on the RUTI [23]. A study by Ormeño et al. showed that there were high rates of antibiotic resistance to the usual antibiotics in *E. coli* causing UTI, which emerged as a risk factor for the development of RUTI [35]. This study demonstrated that there was no significant increase in antimicrobial resistance of most antibiotics between the first and second episodes of HRPFGE *E. coli* RUTI. There were correlations among prior antibiotic therapy within 3 months and subsequent antimicrobial resistance in HRPFGE *E. coli* RUTI, and the use of fluoroquinolones was associated with more antimicrobial resistance of UPEC in the following RUTI. After machine-learning analysis of UTI and wound infections, Stracy et al. suggested that selection for existing resistant strains rather than de novo evolution is the predominant mechanism of treatment-induced emergence of resistance [7].

There are several limitations in our study. First, this was a single-center study with retrospective design and a relatively small sample size was enrolled. Therefore, a multicenter prospective study with a larger sample size is needed to verify the observations of our study. Second, we did not include all important characteristics of patients and *E. coli* in our analyzes. Third, the duration of antibiotic therapy and the severity of UTI were not included in the analysis. Fourth, the determination of genetic relatedness among *E. coli* strains isolated from a single patient was based on molecular typing, not whole genome sequencing.

## Conclusions

This study provides a profile of host and bacterial characteristics of *E. coli* strains in RUTI based on the molecular typing. Compared to the overall genetically different strains, the uropathogens were more virulent in genetically highly related *E. coli* strains in RUTI. Higher bacterial virulence in young age group (< 20 years) and patients with neither anatomical/functional defect nor immune dysfunction suggests that more virulent UPEC strains are needed for the development of RUTI in healthy populations. Prior antibiotic therapy within 3 months could induce subsequent antimicrobial resistance in genetically highly related *E. coli* RUTI.

## Materials and methods

### Sample collection

This is a single-center retrospective cohort study. The study enrolled patients aged 20 years or above who presented with symptoms of UTI in emergency department (ED) or outpatient clinics of National Cheng Kung University Hospital (NCKUH) between August 2009 and December 2010. Data regarding clinical and demographic characteristics, comorbidities, and prescribed medication were collected from the electronic medical record. RUTI was defined as patients had 2 or more infections in 6 months or 3 or more in 12 months during the study period [5]. Each episode of UTI presented with UTI symptoms including pain on urination, lumbago or fever and a bacterial count of more than 10^5^ colony-forming units/mL from a urine specimen (collected from midstream or catheterized urine). The duration between two episodes of *E. coli* RUTI in this study was more than 2 weeks. Anatomical/functional defects included urinary tract obstruction, neurogenic bladder, urolithiasis, urinary tract tumor, vesicoureteral reflux, kidney transplantation, and indwelling catheters or drainage devices; immune dysfunction included diabetes, cirrhosis, malignancy, autoimmune disease, renal failure, and immunosuppression. This study was reviewed and approved by the Institutional Review Board of National Cheng Kung University Hospital, Tainan, Taiwan (B-ER-109-565). All procedures and methods were performed in accordance with the relevant guidelines and regulations.

## DNA extraction and random amplified polymorphic DNA-PCR

Genomic DNA for *E. coli* was prepared using the Qiagen DNeasy Blood and Tissue kit (California, USA), according to the manufacturer’s instructions. Four primers, namely 1247, 1254, 1283, and 1290 [36], were used in RAPD-PCR assay to determine the clonality of *E. coli* isolated from a single patient. RAPD-profiles varying from each other in the positions of up to three bands were considered closely related.

## Pulsed-field gel electrophoresis typing

PFGE of *Xba*I-digested genomic DNA was performed with a CHEF Mapper XA apparatus (Bio-Rad Laboratories, Inc., Hercules, CA, United States) using a 1% agarose gel (Seakem Gold agarose; FMC Bio Products) in 0.5× Tris-Borate-EDTA for 19 h at 14ºC with pulsed times ranging from 5 to 35 s at 6 V/cm. The gels were stained with ethidium bromide and photographed with UV transillumination. PFGE profiles were subjected to data processing using the GelCompar II software, version 2.0 (Unimed Healthcare, Inc., Houston, TX, United States), with a tolerance of 0.9% and an optimization parameter of 0.9%. Strains were considered to be genetically highly-related if they possessed > 85% similarity to the restriction fragment patterns of DNA [10, 37].

## Phylogenetic analysis

The phylogenetic grouping of the *E. coli* isolates was determined by an algorithm of PCR-based method proposed by Clermont *et al* [38]. *E. coli* isolates were assigned to one of the four main phylogenetic groups (A, B1, B2, and D) according to the presence of *chuA*, *yjaA*, and the DNA fragment TSPE4.C2 [12, 39].

## Detection of virulence genes

Sixteen uropathogenic virulence factor genes of *E. coli* were determined using PCR. Primer pairs specific for K1 capsule gene (*neuA*), 3 PapG adhesion genes (*papG* class I to III) of P-fimbriae, and genes for type 1 fimbrial adhesins (*fimH*), S-/F1C-fimbriae (*sfa*/*foc*), afimbrial adhesins (*afa*), iron regulated gene A homologue adhesins (*iha*), hemolysin (*hlyA*), cytotoxic necrotizing factor 1 (*cnf1*), catecholate siderophore receptor (*iroN*), aerobactin receptor (*iutA*), outer membrane protease T (*ompT*), and uropathogenic specific protein (*usp*) have been described previously [13, 16, 18, 40–42]. Positive and negative control clinical isolates derived from our previous study [42] for each gene were also used in each assay.

## Determination of antimicrobial susceptibility

The minimum inhibitory concentrations (MICs) to flomoxef (FLO), ampicillin-sulbactam (SAM), piperacillin/tazobactam (TZP), cefazolin (CZ), cefuroxime (CXM), cefmetazole (CMZ), ceftazidime (CAZ), ceftriaxone (CRO), cefoperazone/sulbactam (CFS), cefepime (FEP), ertapenem (ETP), imipenem (IPM), amikacin (AN), gentamicin (GM), ciprofloxacin (CIP), levofloxacin (LVX), tigecycline (TGC), and trimethoprim/sulfamethoxazole (SXT) by Vitek 2 testing using software version 5.04 and the AST-GN69 and AST-XN06 cards, according to the manufacturer’s instructions. *E. coli* ATCC 25922 was used as a quality control strain. The interpretation of resistance was determined according to the recommendations of the Clinical and Laboratory Standards Institute (CLSI) guideline [17, 43].

### Statistical analysis

The Chi-square test or Fisher’s exact test (two-tailed) was used for the comparison of categorical factors, whereas the Wilcoxon rank-sum test or Pearson’s Chi-squared test was used for the comparison of continuous factors between groups. A *p* value < 0.05 was considered to be statistically significant. All statistical analyses were performed using JMP software version 7.0 (SAS Institute Inc., Cary, NC, USA).

## Electronic Supplementary Material

Below is the link to the electronic supplementary material

Supplementary Material 1

Supplementary Material 2

## Data Availability

The datasets used and/or analyzed during the current study available from the corresponding author on reasonable request.
