# Supplementary material for: Characterization of host and escherichia coli strains causing recurrent urinary tract infections based on molecular typing
Source: BMC Microbiol. 2023 Mar 30;23:90. doi: 10.1186/s12866-023-02820-1 (PMC10061793; doi:10.1186/s12866-023-02820-1)
Supplement: Supplementary file 1 — Supplementary Material 1 [file 12866_2023_2820_MOESM1_ESM.ppt]

## Slide 1
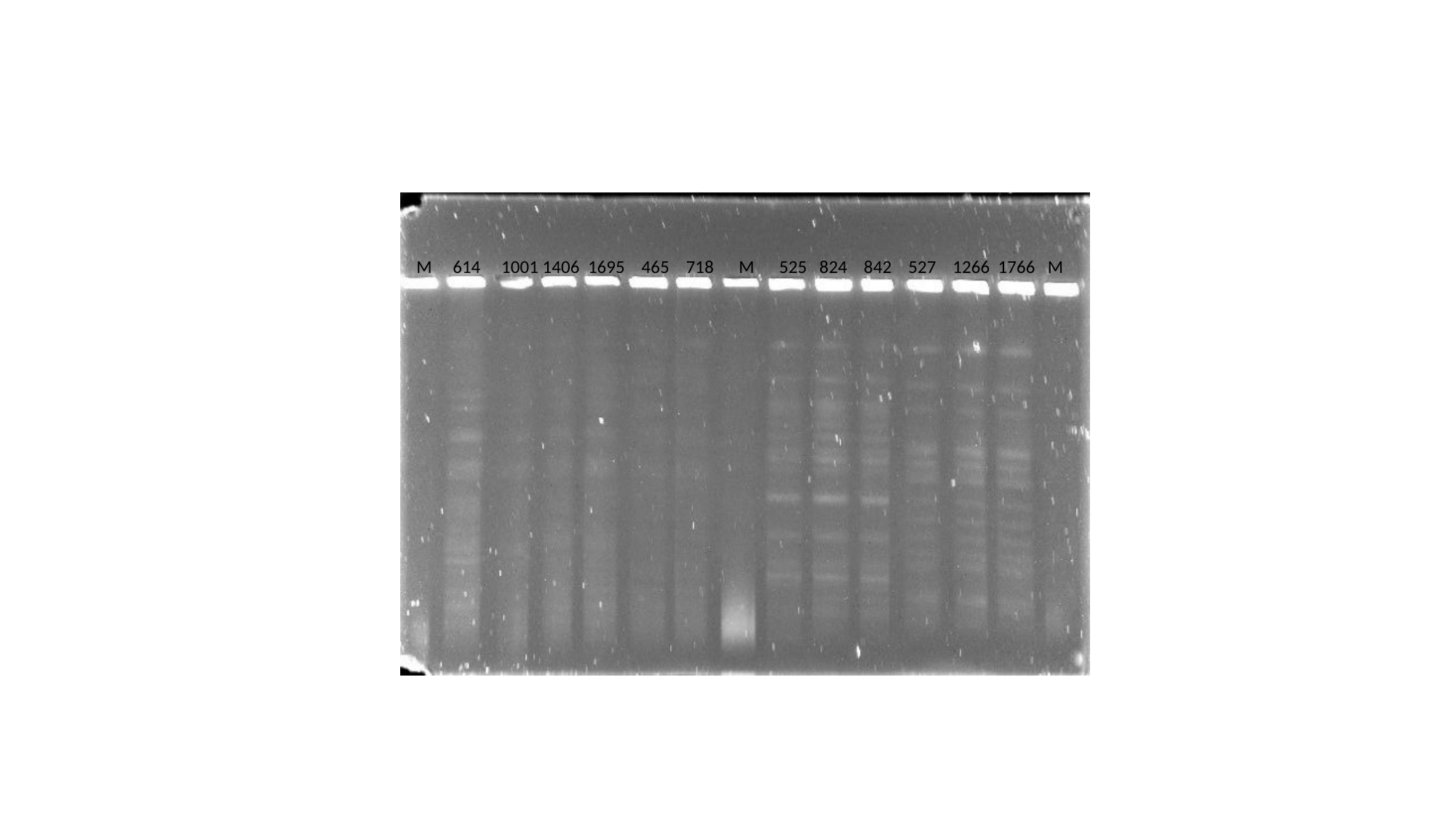

M 614 1001 1406 1695 465 718 M 525 824 842 527 1266 1766 M

## Slide 2
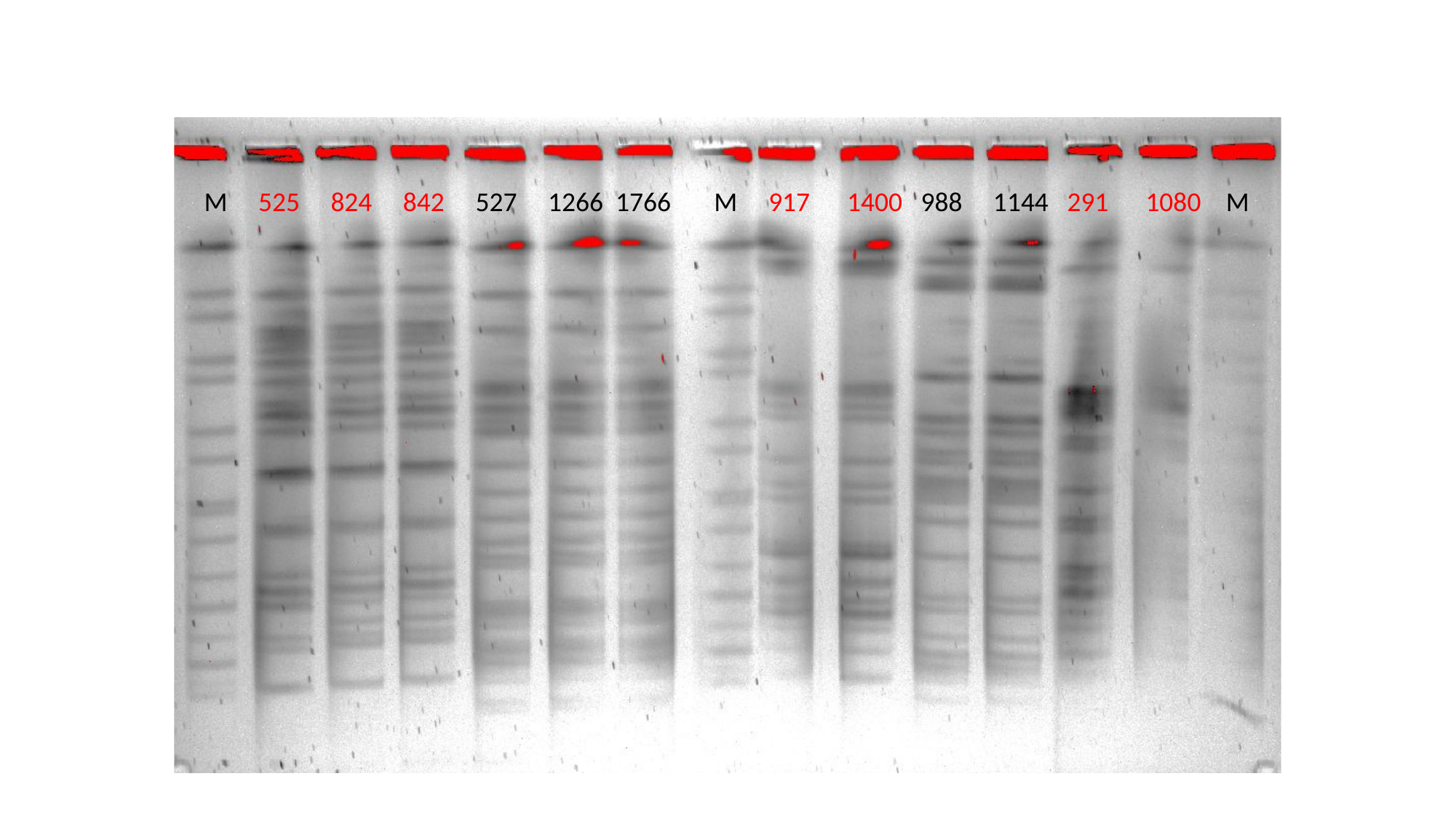

M 525 824 842 527 1266 1766 M 917 1400 988 1144 291 1080 M

## Slide 3
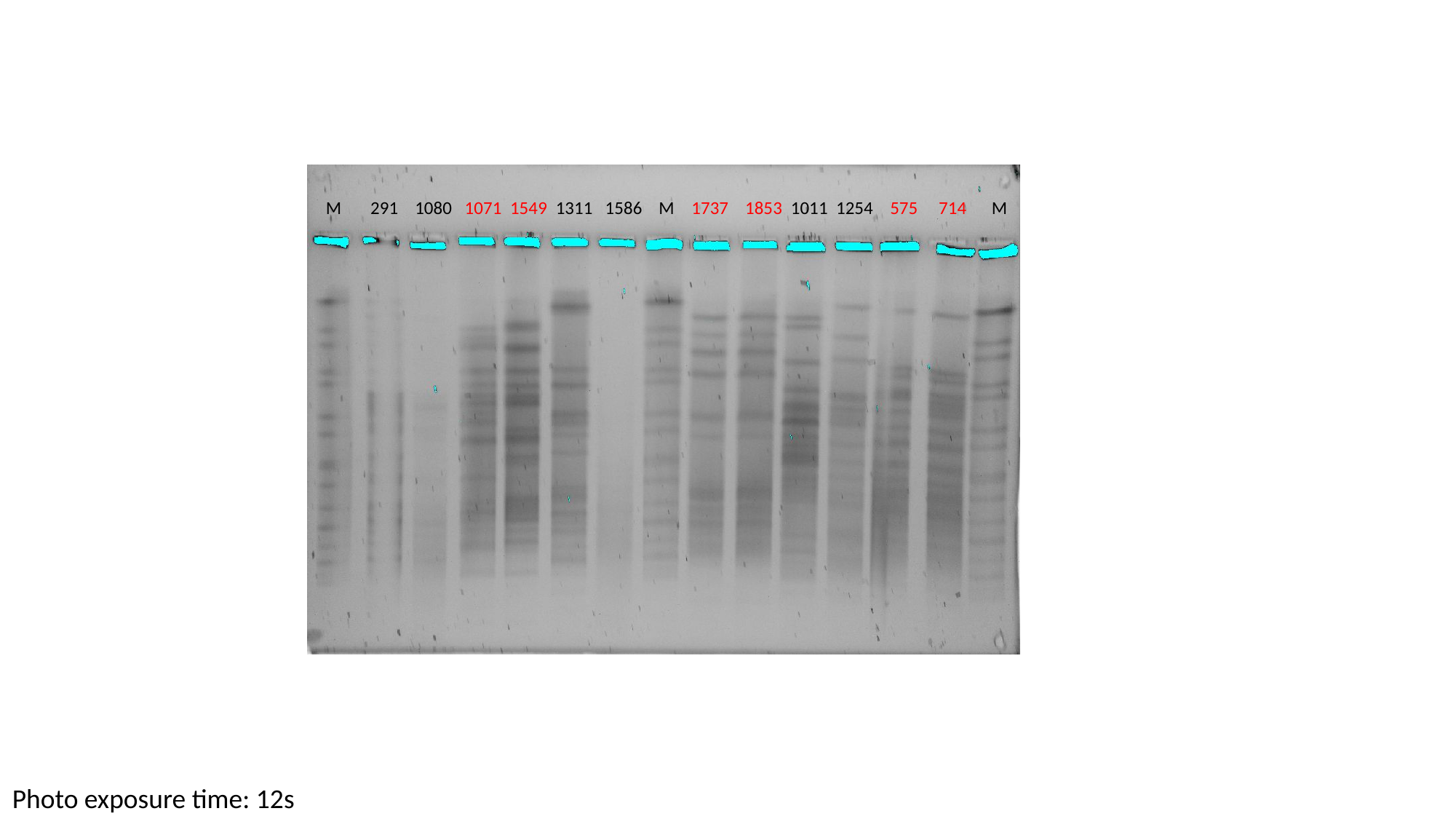

M 291 1080 1071 1549 1311 1586 M 1737 1853 1011 1254 575 714 M
Photo exposure time: 12s

## Slide 4
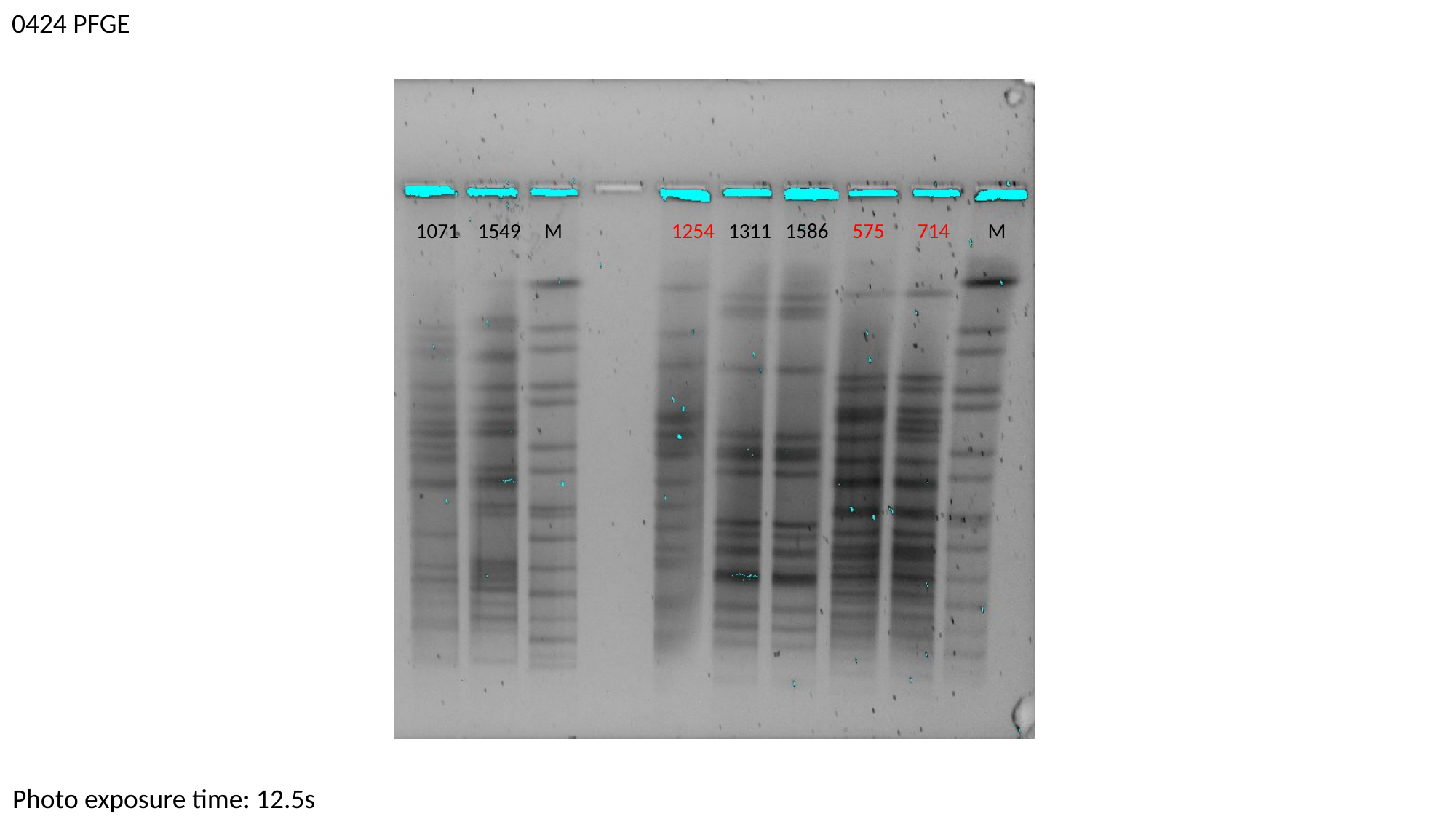

0424 PFGE
1071 1549 M 1254 1311 1586 575 714 M
Photo exposure time: 12.5s

## Slide 5
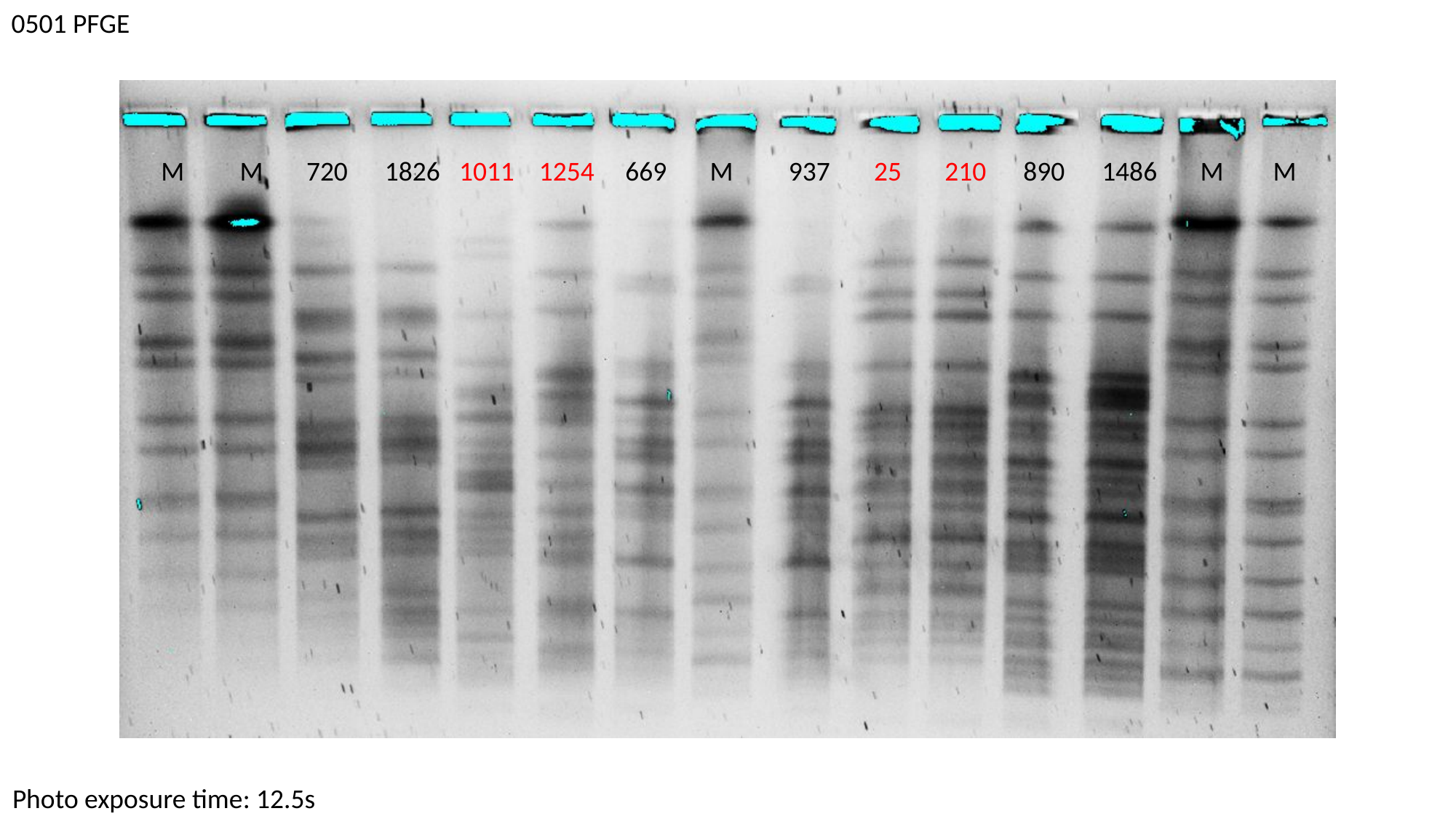

0501 PFGE
M M 720 1826 1011 1254 669 M 937 25 210 890 1486 M M
Photo exposure time: 12.5s

## Slide 6
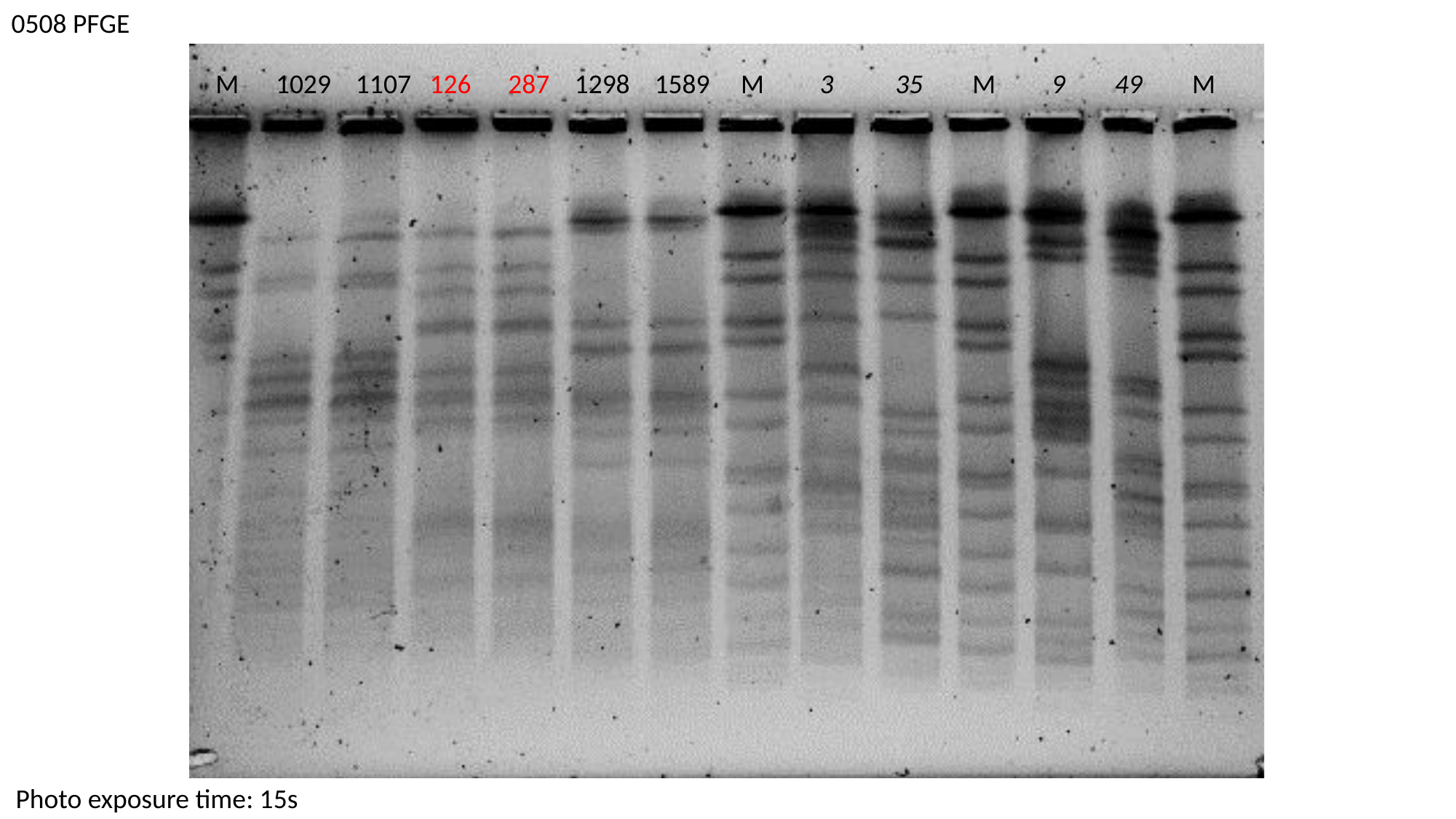

0508 PFGE
M 1029 1107 126 287 1298 1589 M 3 35 M 9 49 M
Photo exposure time: 15s

## Slide 7
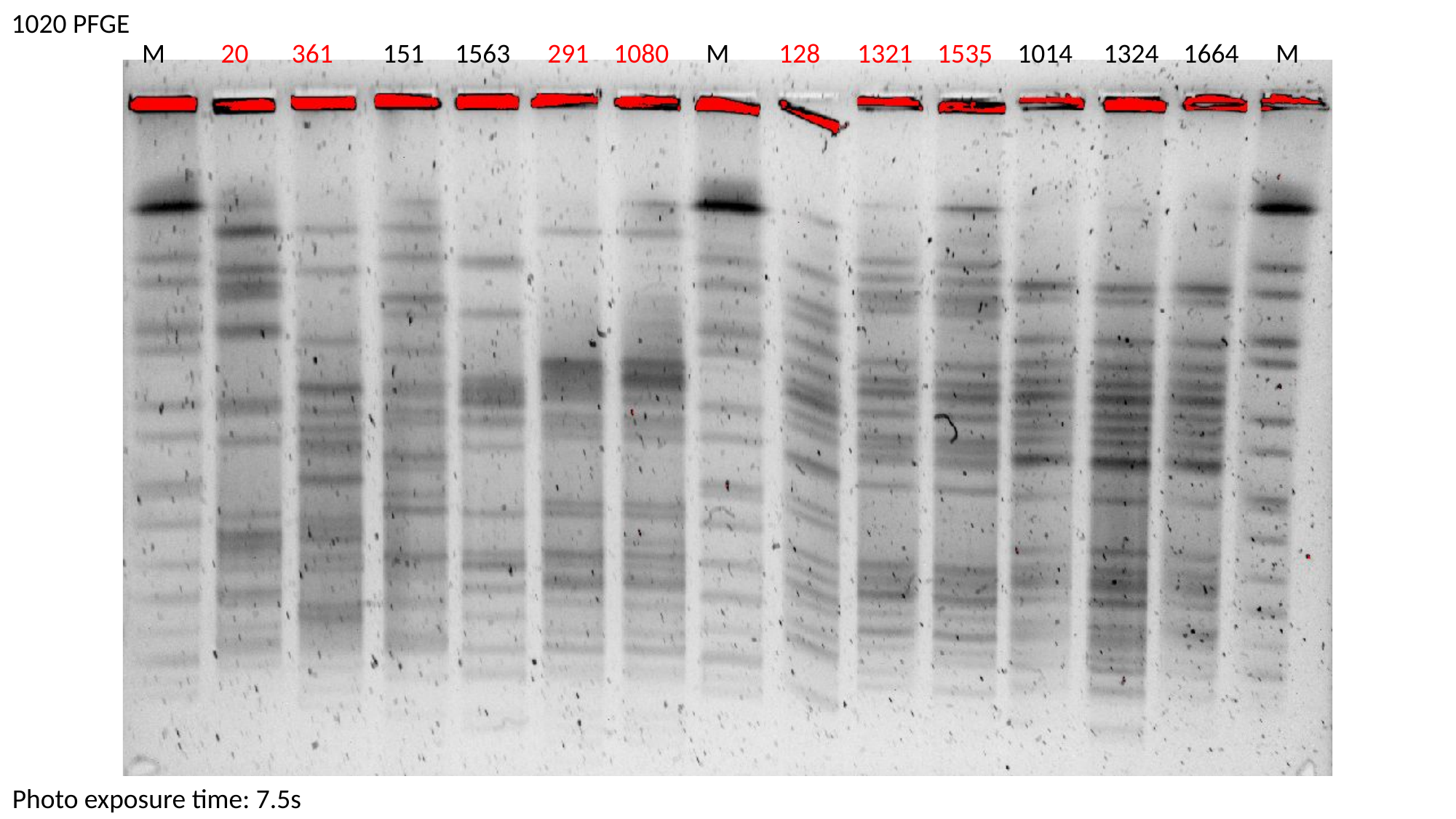

1020 PFGE
M 20 361 151 1563 291 1080 M 128 1321 1535 1014 1324 1664 M
Photo exposure time: 7.5s

## Slide 8
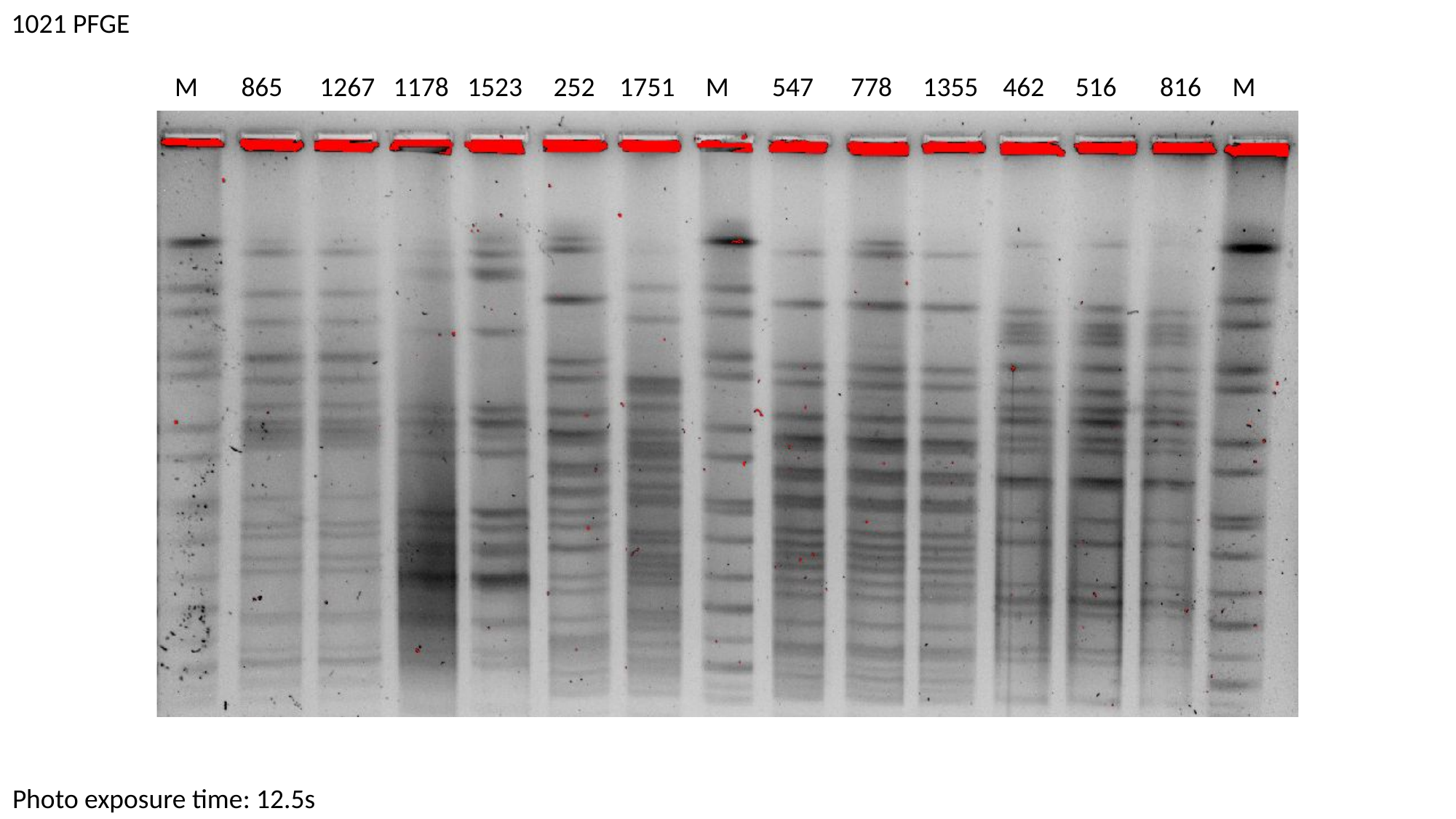

1021 PFGE
M 865 1267 1178 1523 252 1751 M 547 778 1355 462 516 816 M
Photo exposure time: 12.5s

## Slide 9
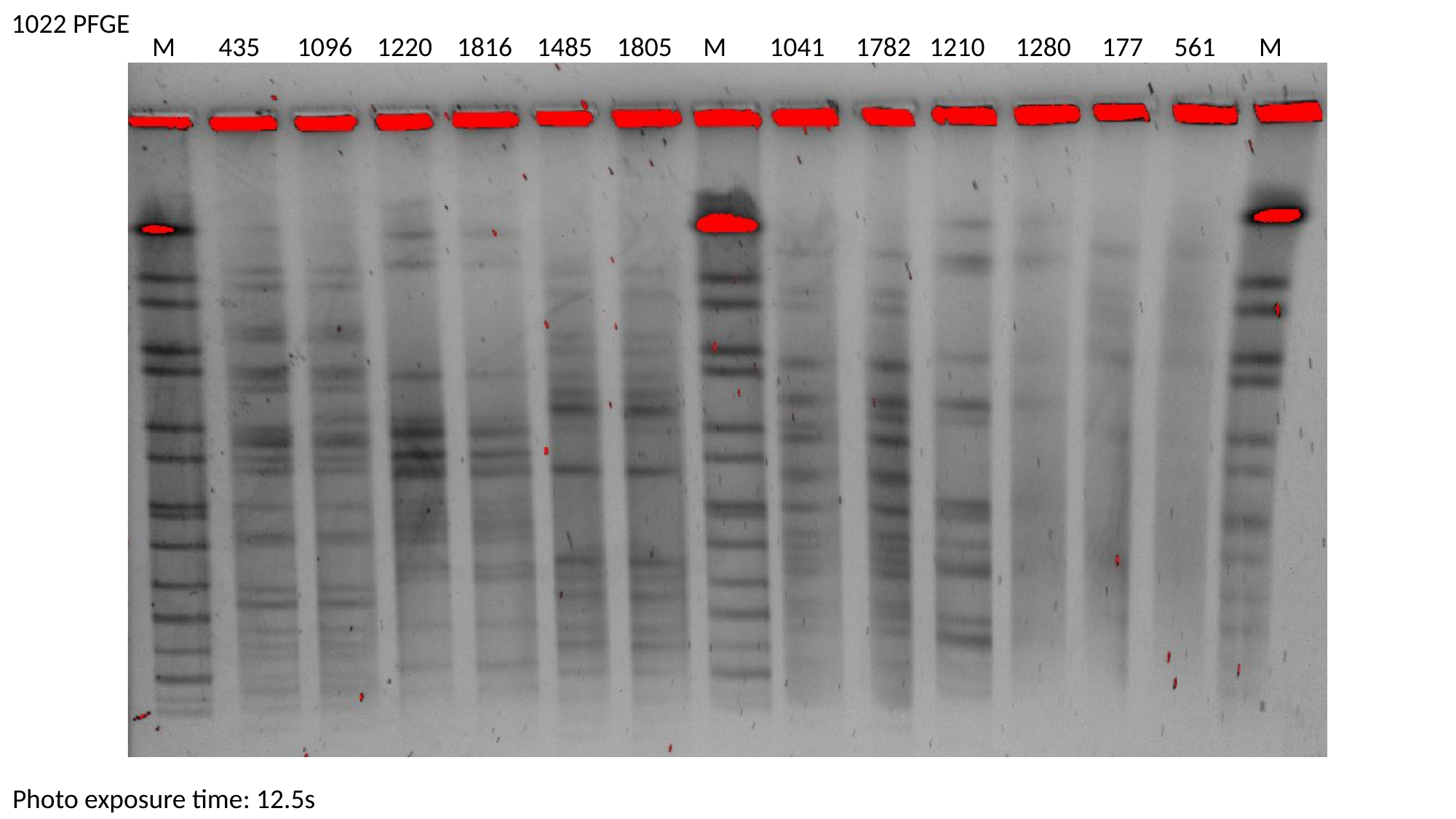

1022 PFGE
M 435 1096 1220 1816 1485 1805 M 1041 1782 1210 1280 177 561 M
Photo exposure time: 12.5s

## Slide 10
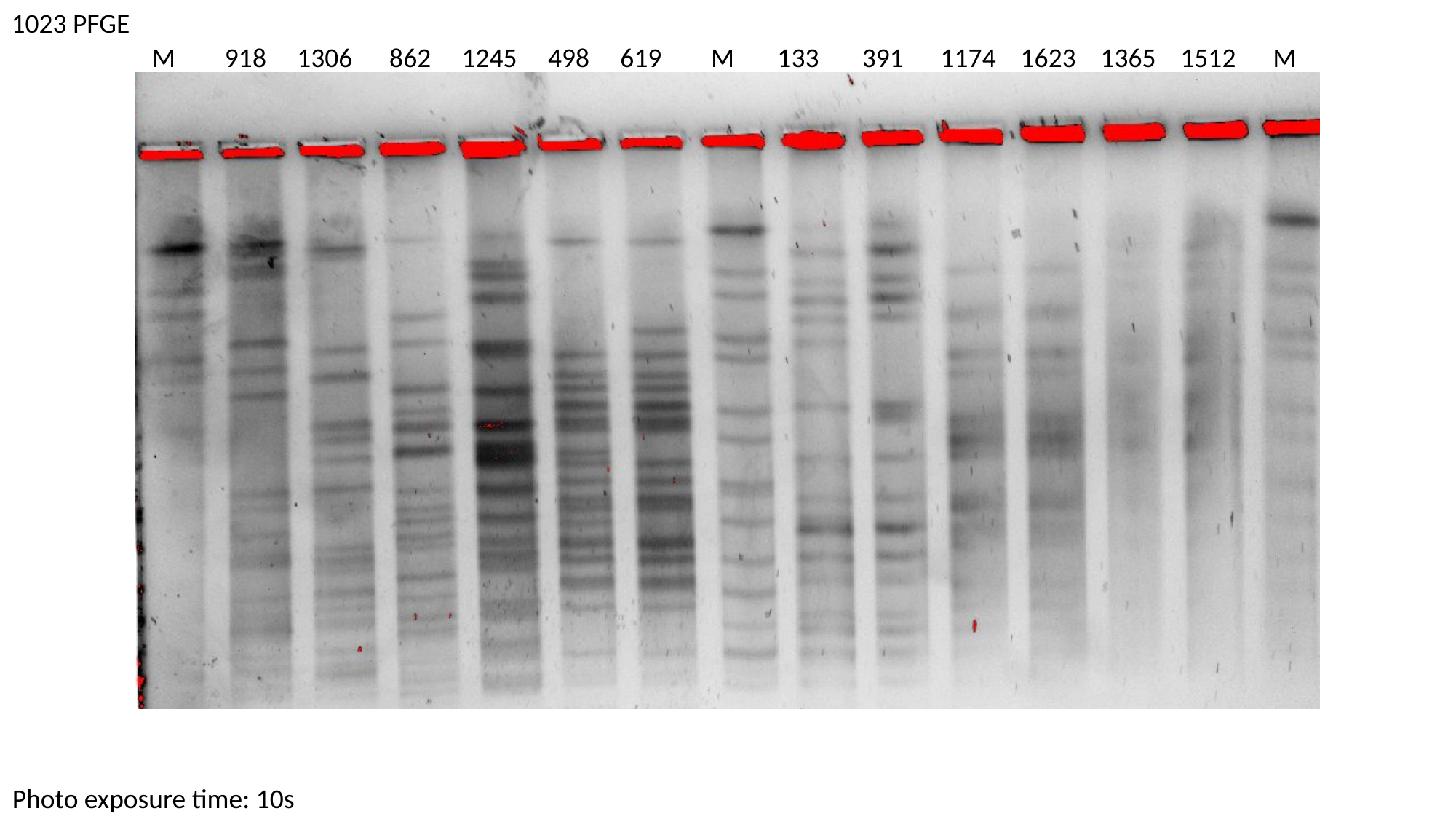

1023 PFGE
M 918 1306 862 1245 498 619 M 133 391 1174 1623 1365 1512 M
Photo exposure time: 10s

## Slide 11
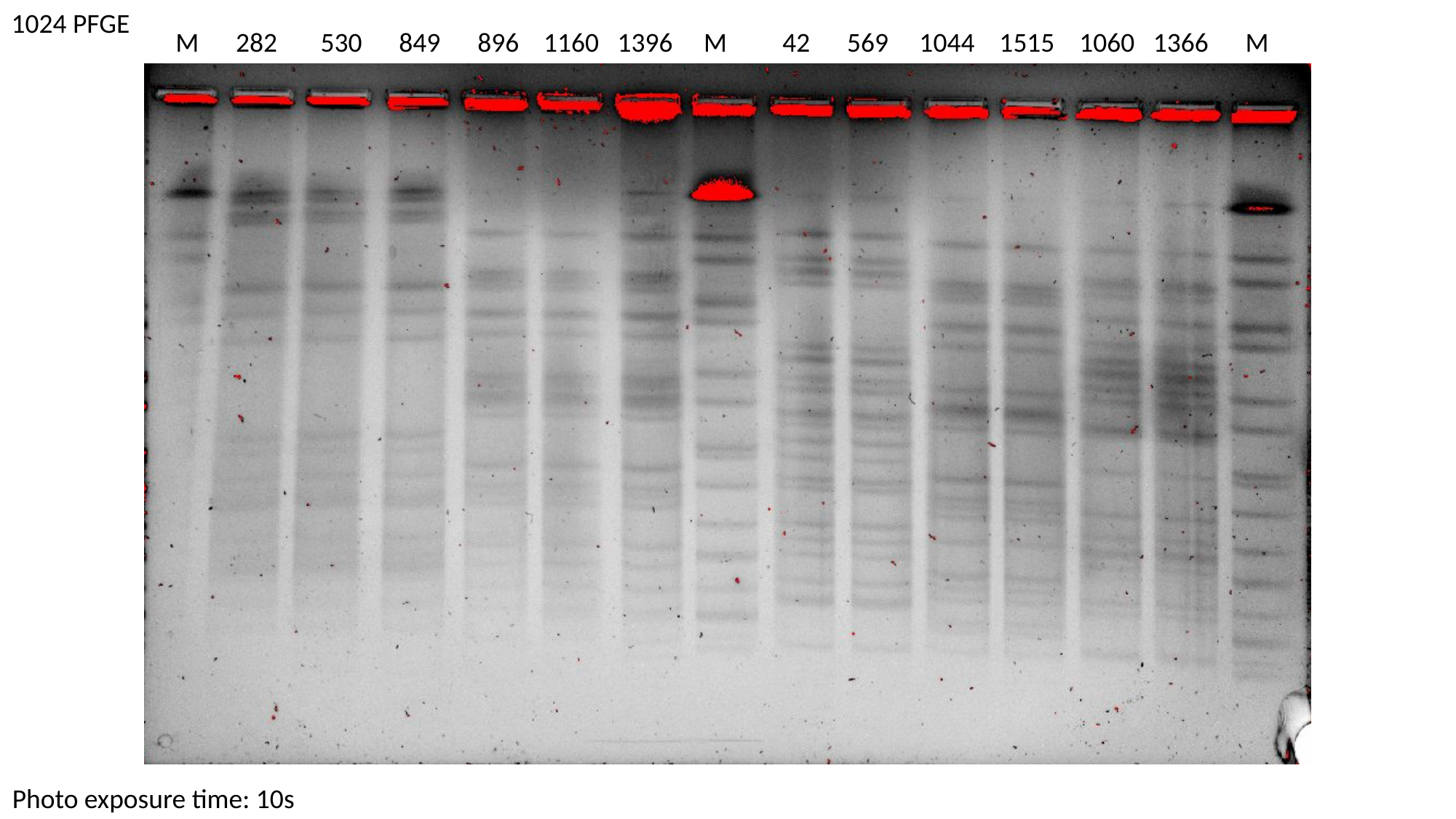

1024 PFGE
M 282 530 849 896 1160 1396 M 42 569 1044 1515 1060 1366 M
Photo exposure time: 10s

## Slide 12
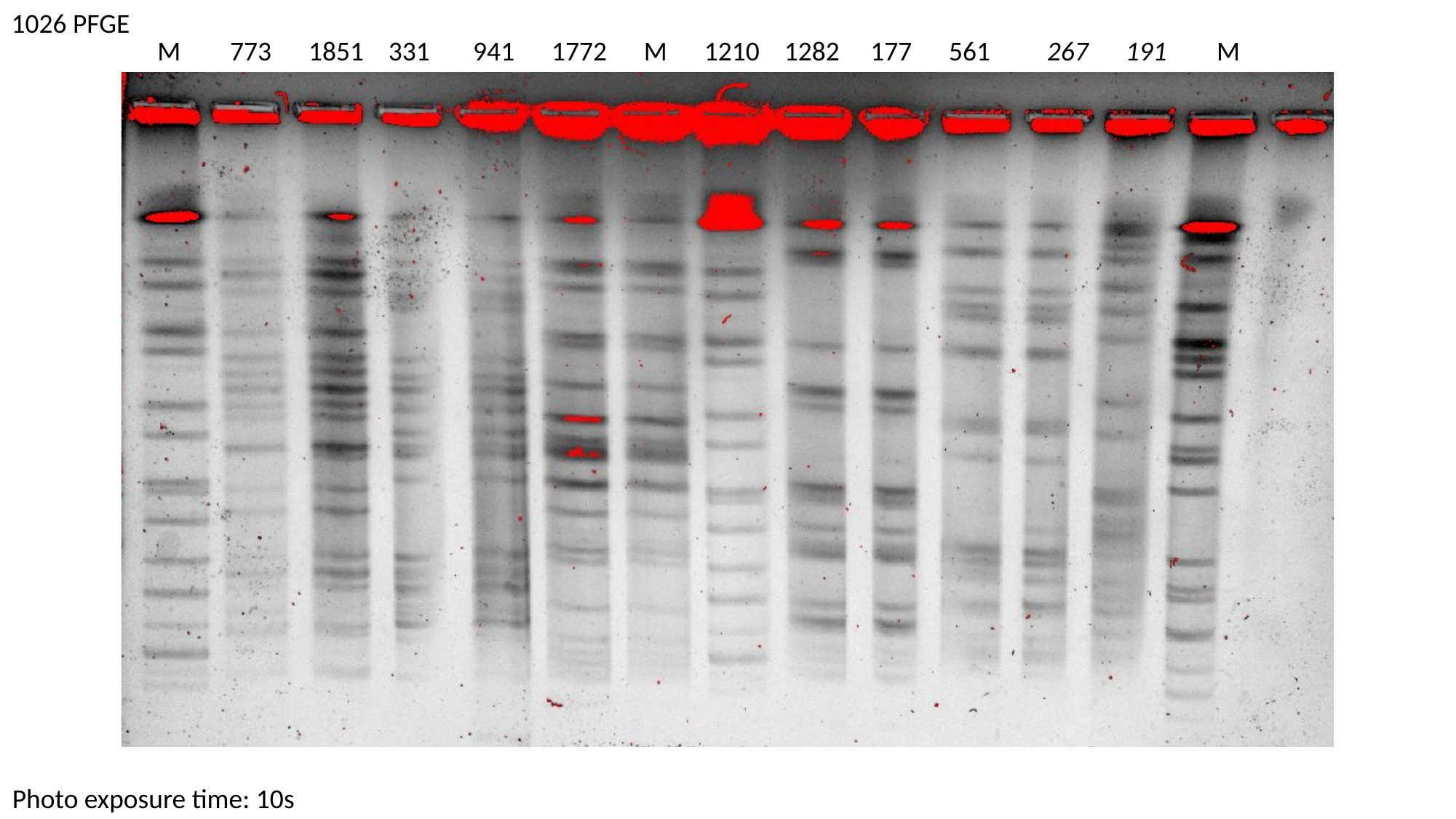

1026 PFGE
M 773 1851 331 941 1772 M 1210 1282 177 561 267 191 M
Photo exposure time: 10s

## Slide 13
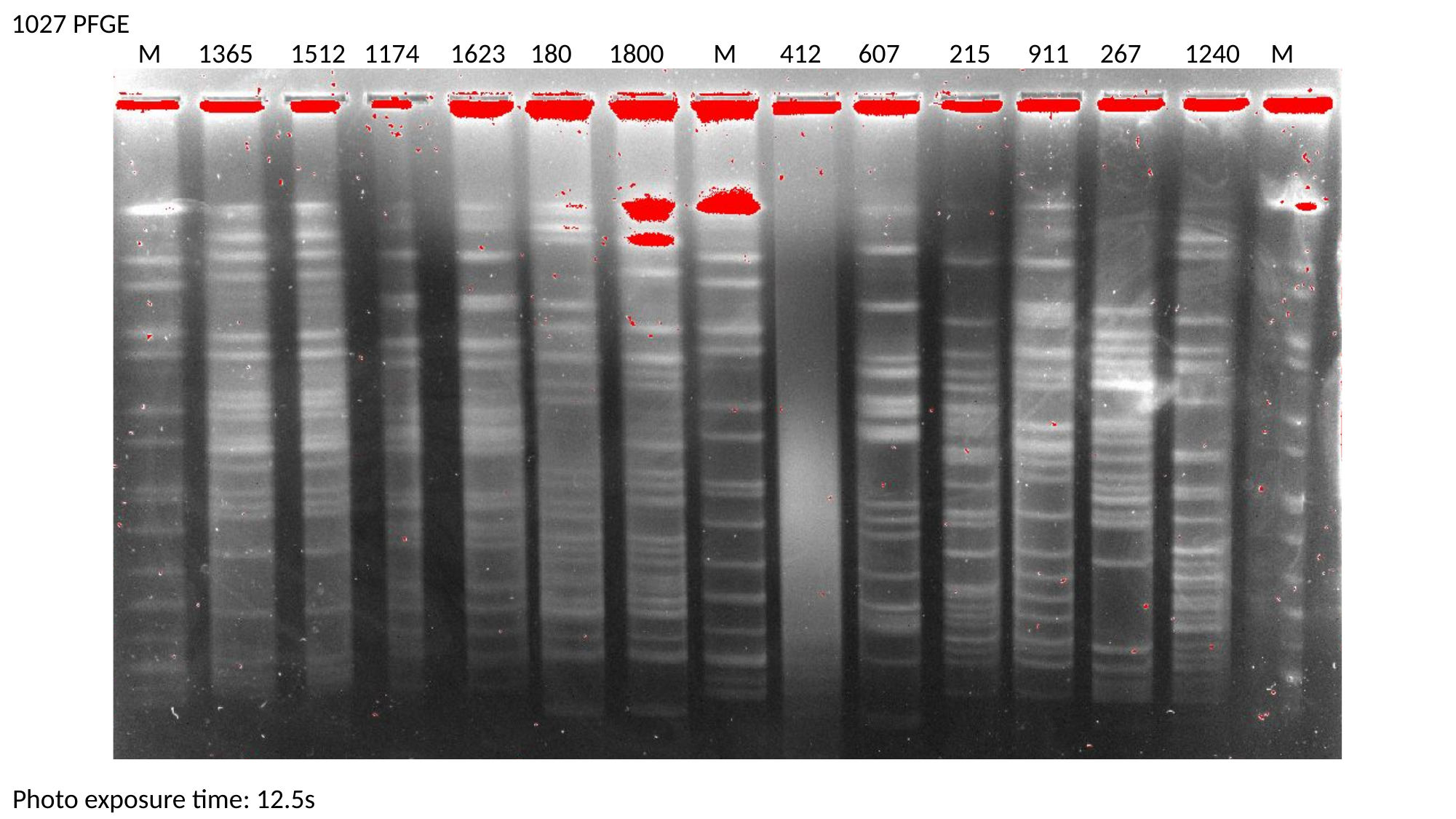

1027 PFGE
M 1365 1512 1174 1623 180 1800 M 412 607 215 911 267 1240 M
Photo exposure time: 12.5s
