# Supplementary material for: Characterization of host and escherichia coli strains causing recurrent urinary tract infections based on molecular typing
Source: BMC Microbiol. 2023 Mar 30;23:90. doi: 10.1186/s12866-023-02820-1 (PMC10061793; doi:10.1186/s12866-023-02820-1)
Supplement: Supplementary file 2 — Supplementary Material 2 [file 12866_2023_2820_MOESM2_ESM.doc]

**Supplementary Information**

**Table S1**. Bacterial characteristics in relation to gender in 99 patients with recurrent urinary tract infection (first episode)

| Characteristic | Male (n = 26)  n (%) | Female (n = 73)  n (%) | *P*-value |
| --- | --- | --- | --- |
| Phylogenetic group |  |  | 0.1807 |
| A | 0 | 4 (5) |  |
| B1 | 0 | 10 (14) |  |
| B2 | 17 (65) | 38 (52) |  |
| D | 8 (31) | 20 (27) |  |
| Untypable | 1 (4) | 1 (1) |  |
| Virulence factor |  |  |  |
| *neuA* | 5 (19) | 16 (22) | 1.0000 |
| *papG I* | 0 | 0 | -- |
| *papG II* | 6 (23) | 14 (19) | 0.7769 |
| *papG III* | 2 (8) | 12 (16) | 0.3440 |
| *sfa* | 2 (8) | 4 (5) | 0.6515 |
| *foc* | 1 (4) | 6 (8) | 0.6721 |
| *cnf1* | 5 (19) | 10 (14) | 0.5310 |
| *aer* | 19 (73) | 48 (66) | 0.6271 |
| *usp* | 16 (62) | 40 (55) | 0.6474 |
| *iha* | 11 (42) | 18 (25) | 0.1310 |
| *ompT* | 20 (77) | 55 (75) | 1.0000 |
| *afa* | 8 (31) | 36 (49) | 0.1142 |
| *iroN* | 8 (31) | 27 (37) | 0.6386 |
| *fimH* | 23 (88) | 71 (97) | 0.1117 |
| *hlyA* | 7 (27) | 12 (16) | 0.2571 |
| *sat* | 11 (42) | 16 (22) | 0.0708 |

**Table S2.** Bacterial characteristics in relation to age in 99 patients with recurrent urinary tract infection (first episode)

| Characteristic | Age <20 years  (n = 11)  n (%) | Age 20-64 years  (n = 31)  n (%) | Age ≥65 years  (n = 57)  n (%) | *P*-value |
| --- | --- | --- | --- | --- |
| Phylogenetic group |  |  |  | 0.6125 |
| A | 0 | 1 (3) | 3 (5) |  |
| B1 | 1 (9) | 5 (16) | 4 (7) |  |
| B2 | 5 (45) | 19 (61) | 31 (54) |  |
| D | 5 (45) | 6 (19) | 17 (30) |  |
| Untypable | 0 | 0 | 2 (4) |  |
| Virulence factor |  |  |  |  |
| *neuA* | 0 | 6 (19) | 15 (26) | 0.1413 |
| *papG I* | 0 | 0 | 0 | -- |
| *papG II* | 1 (9) | 6 (19) | 13 (23) | 0.5781 |
| *papG III* | 2 (18) | 4 (13) | 8 (14) | 0.9105 |
| *sfa* | 0 | 1 (3) | 5 (9) | 0.3900 |
| *foc* | 3 (27) | 2 (6) | 2 (4) | 0.0188 |
| *cnf1* | 4 (36) | 6 (19) | 5 (9) | 0.0478 |
| *aer* | 6 (55) | 23 (74) | 38 (67) | 0.4734 |
| *usp* | 5 (45) | 19 (61) | 32 (56) | 0.6575 |
| *iha* | 2 (18) | 7 (23) | 20 (35) | 0.3240 |
| *ompT* | 8 (73) | 23 (74) | 44 (77) | 0.9230 |
| *afa* | 3 (27) | 17 (55) | 24 (42) | 0.2470 |
| *iroN* | 3 (27) | 12 (39) | 20 (35) | 0.7910 |
| *fimH* | 11 (100) | 31 (100) | 52 (91) | 0.1437 |
| *hlyA* | 4 (36) | 4 (13) | 11 (19) | 0.2366 |
| *sat* | 2 (18) | 7 (23) | 18 (32) | 0.5129 |

**Table S3.** Antimicrobial susceptibility in 41 patients with recurrent urinary tract infection related to highly related PFGE *Escherichia coli* strains (91 episodes)

| Antibiotic | Susceptible  n (%) | Intermediate  n (%) | Resistance  n (%) |
| --- | --- | --- | --- |
| Flomoxef | 72 (79) | 13 (14) | 6 (7) |
| Ampicillin/sulbactam | 26 (29) | 7 (8) | 58 (64) |
| Piperacillin/tazobactam | 71 (78) | 10 (11) | 10 (11) |
| Cefazolin | 45 (49) | 0 | 46 (51) |
| Cefuroxime | 42 (46) | 6 (7) | 43 (47) |
| Cefmetazole | 68 (75) | 8 (9) | 15 (16) |
| Ceftriaxone | 45 (49) | 1 (1) | 45 (49) |
| Ceftazidime | 54 (59) | 3 (3) | 34 (37) |
| Cefoperazone/sulbactam | 81 (89) | 4 (4) | 6 (7) |
| Cefepime | 73 (80) | 9 (10) | 9 (10) |
| Ertapenem | 91 (100) | 0 | 0 |
| Imipenem | 91 (100) | 0 | 0 |
| Gentamicin | 55 (60) | 1 (1) | 35 (38) |
| Amikacin | 90 (99) | 0 | 1 (1) |
| Ciprofloxacin | 34 (37) | 0 | 57 (63) |
| Levofloxacin | 31 (34) | 4 (4) | 56 (62) |
| Tigecycline | 91 (100) | 0 | 0 |
| Trimethoprim/sulfamethoxazole | 49 (54) | 0 | 42 (46) |

PFGE: Pulsed-field gel electrophoresis

**Table S4.** Antimicrobial susceptibility in recurrent urinary tract infection related to highly related PFGE *Escherichia coli* strains (41 patients, 91 episodes)

| Antibiotic | Susceptible,  first episode  (n = 41)  n (%) | Susceptible,  second episode  (n = 41)  n (%) | Susceptible,  third episode  (n = 8)  No. (%) |
| --- | --- | --- | --- |
| Flomoxef | 33 (80) | 32 (78) | 6 (75) |
| Ampicillin/sulbactam | 10 (24) | 13 (32) | 3 (38) |
| Piperacillin/tazobactam | 33 (80) | 32 (78) | 5 (63) |
| Cefazolin | 22 (54) | 21 (51) | 2 (25) |
| Cefuroxime | 21 (51) | 19 (46) | 2 (25) |
| Cefmetazole | 32 (78) | 30 (73) | 5 (63) |
| Ceftriaxone | 22 (54) | 21 (51) | 2 (25) |
| Ceftazidime | 25 (61) | 25 (61) | 4 (50) |
| Cefoperazone/sulbactam | 37 (90) | 36 (88) | 7 (88) |
| Cefepime | 33 (80) | 34 (83) | 6 (75) |
| Ertapenem | 41 (100) | 41 (100) | 8 (100) |
| Imipenem | 41 (100) | 41 (100) | 8 (100) |
| Gentamicin | 25 (61) | 26 (63) | 4 (50) |
| Amikacin | 41 (100) | 40 (98) | 8 (100) |
| Ciprofloxacin | 16 (39) | 16 (39) | 2 (25) |
| Levofloxacin | 15 (37) | 14 (34) | 2 (25) |
| Tigecycline | 41 (100) | 41 (100) | 8 (100) |
| Trimethoprim/sulfamethoxazole | 23 (56) | 19 (46) | 6 (75) |

PFGE: pulsed-field gel electrophoresis

**Table S5.** Correlations among prior antibiotic therapy within 3 months and subsequent antimicrobial resistance in 91 episodes of recurrent urinary tract infection related to highly related PFGE *Escherichia coli* strains (41 patients)

| Prior antibiotic therapy within 3 months | Antimicrobial resistance | | | | | | | | | | | |
| --- | --- | --- | --- | --- | --- | --- | --- | --- | --- | --- | --- | --- |
|  | FLO | SAM | TZP | CZ | CXM | CAZ | ETP | GM | AN | CIP | LVX | SXT |
| Any antibiotic | 0.1606, 0.1373 | 0.1732, 0.0781 | 0.2496, 0.0279 | 0.2944, 0.0043 | 0.2769, 0.0065 | 0.3041, 0.0043 | -- | 0.1253,  0.2221 | 0.0742, 0.5791 | 0.1703, 0.0879 | 0.2613, 0.0082 | 0.1949, 0.0577 |
| FLO | 0.2069,  0.6055 | 0.2480,  0.5248 | 0.2131,  0.5936 | 0.3590,  0.4945 | 0.3763,  0.2774 | 0.3090,  0.4052 | -- | 0.3035,  0.4159 | 0.0447,  0.9156 | 0.4249,  0.1929 | 0.4445,  0.1618 | 0.0574,  0.3519 |
| Piperacillin or TZP | 0.1954,  0.1365 | 0.1319,  0.3839 | 0.1838,  0.1650 | 0.2897,  0.0528 | 0.2651,  0.0783 | 0.3657,  0.0115 | -- | 0.1389,  0.3221 | 0.0574,  0.7716 | 0.2000,  0.1890 | 0.1749,  0.2504 | 0.2005,  0.1627 |
| 1st generation cephalosporins (CZ or cephalexin) | 0.0866,  0.3331 | 0.0361,  0.6877 | 0.1109,  0.2141 | 0.0490,  0.5859 | 0.0387,  0.6709 | 0.0831  0.3554 | -- | 0.1386,  0.1218 | 0.0954,  0.3839 | 0.0954,  0.2876 | 0.0100,  0.8984 | 0.1526,  0.0892 |
| 2nd or 3rd generation cephalosporins | 0.2105,  0.0197 | 0.1913,  0.0441 | 0.1425,  0.1157 | 0.1145,  0.2149 | 0.0721,  0.4359 | 0.1637  0.0746 | -- | 0.0557,  0.5472 | 0.0860,  0.4590 | 0.0825,  0.3747 | 0.1253,  0.1790 | 0.1311,  0.1548 |
| Amoxicillin or SAM | 0.2698,  0.1282 | 0.3240,  0.0611 | 0.1425,  0.2669 | 0.0000,  0.9740 | 0.0316,  0.8193 | 0.0762,  0.5733 | -- | 0.1233,  0.3778 | 0.0583,  0.7549 | 0.0000,  0.9933 | 0.1975,  0.1778 | 0.1319,  0.3315 |
| Fluoroquinolones | 0.2105,  0.0197 | 0.0510,  0.5786 | 0.1900,  0.0354 | 0.3217,  0.0006 | 0.3662,  0.0001 | 0.2876,  0.0018 | -- | 0.0557,  0.5472 | 0.0860,  0.4590 | 0.3020,  0.0016 | 0.2612,  0.0063 | 0.0100,  0.9191 |
| Aminoglycosides | 0.2311,  0.1428 | 0.2901,  0.1958 | 0.2205,  0.1663 | 0.0000,  0.9821 | 0.0894  0.8746 | 0.0671,  0.6973 | -- | 0.4845,  0.0115 | 0.0529,  0.8293 | 0.0917,  0.5931 | 0.3231,  0.1415 | 0.4416  0.0272 |
| SXT | 0.2330, 0.3656 | 0.0361, 0.8527 | 0.2398, 0.3499 | 0.1192, 0.5442 | 0.1418, 0.4686 | 0.0520, 0.7928 | -- | 0.0436, 0.8225 | 0.0510, 0.8527 | 0.2035, 0.2860 | 0.2278, 0.2257 | 0.0894, 0.6506 |

Note: Intermediate was considered as resistance in antimicrobial susceptibility in the correlation analysis.

Data are expressed as r (Pearson’s correlation coefficient) and P value

Pearson’s correlation test was used to measure the correlations among parameters

PFGE, pulsed-field gel electrophoresis; FLO, Flomoxef; SAM, Ampicillin/sulbactam; TZP, Piperacillin/tazobactam; CZ, Cefazolin; CXM, Cefuroxime; CAZ, Ceftazidime; ETP, Ertapenem; GM, Gentamicin; AN, Amikacin; CIP, Ciprofloxacin; LVX, Levofloxacin; SXT, Trimethoprim/sulfamethoxazole; MX, Moxifloxacin
